# Validity of serum adenosine deaminase in diagnosis of tuberculosis

**DOI:** 10.11604/pamj.2013.15.133.2100

**Published:** 2013-08-14

**Authors:** Aliasghar Farazi, Ayda Moharamkhani, Masoome Sofian

**Affiliations:** 1 Department of Infectious Diseases, School of Medicine, Arak University of Medical Sciences, Arak, Iran; 2 Tuberculosis and Pediatrics Infectious Diseases Research Center, Arak University of Medical Sciences, Arak, Iran; 3 Student of Medicine, school of medicine,Arak, Iran

**Keywords:** Adenosine deaminase, sensitivity, specificity, Tuberculosis

## Abstract

**Introduction:**

Tuberculosis is one of the most important infectious causes of death worldwide. Ziehl-Neelsen staining of sputum has high specificity in tuberculosis endemic countries, but modest sensitivity which varies among laboratories. This study was set up to investigate the diagnostic value of serum Adenosine deaminase in diagnosis of tuberculosis.

**Methods:**

In a cross sectional and prospective study Serums of 200 patients of positive sputum smear, negative sputum smear, extra-pulmonary tuberculosis and bacterial community acquired pneumonia collected from March 2011 to May 2012 were evaluated. The data were analyzed using SPSS software and P-value of <0.05 was considered significant.

**Results:**

A total of 200 subjects were included in the study designed in four groups. In cut-off value of ≥24 U/l for ADA in smear positive patients defined the sensitivity, specificity and positive predictive value 12%, 98% and 86% respectively. In smear negative patients defined the 6%, 98% and 75%, and in extra-pulmonary tuberculosis patients defined the sensitivity 14%, 98% and 88% respectively.

**Conclusion:**

This study indicated that measurement of serum ADA level do not have enough sensitivity to assist in the diagnoses of tuberculosis patients from other respiratory diseases and not evaluated perform well enough to replace sputum smear microscopy. Thus, this tests have little role in the diagnosis of pulmonary tuberculosis.

## Introduction

Tuberculosis is a major cause of morbidity and mortality throughout the world. One-third of the world's population is infected with the TB bacillus [1]. The global tuberculosis epidemic results in nearly two million deaths and nine million new cases of the disease per year,95% in developing countries [2]. Once infected active disease develops in about 10% of cases usually within 1-2 years after exposure from TB [3]. The remainder stay in a state of latent tuberculosis infection (LTBI), which can reactivate at a later stage, particularly if the individual is elderly or becomes immune compromised. The simplest rapid method is the detection of acid-fast bacilli by microscopy. However, 40 to 60% of patients with pulmonary disease and about 75% of patients with extra-pulmonary disease are smear negative, and in this situation even contemporary culture methods take several weeks to become positive where the diagnosis of tuberculosis relies on the identification of acid-fast bacilli on unprocessed sputum smears using conventional Ziehl-Neelsen staining [4–6]. Acid-fast staining has high specificity in tuberculosis endemic countries, but modest sensitivity which varies among laboratories (range 20% to 80%) [7, 8]. Moreover, the sensitivity is poor for paucibacillary disease (e.g., pediatric and HIV associated tuberculosis) [9]. Thus, the development of rapid and accurate new diagnostic tools is imperative. There are contradictory reports about the diagnostic value of serum adenosine deaminase in tuberculosis. Adenosine deaminase is involved in the propagation and differentiation of various lymphocytes, particularly T-lymphocytes, so that estimation of its level of activity in various body fluids has been used in the diagnosis of tuberculous effusions especially pleural forms [10]. This study was set up to investigate the diagnostic value of serum Adenosin deaminase in diagnosis of tuberculosis because rapid and accurate diagnosis is an important element of TB treatment and control.

## Methods

In a cross sectional and prospective study Serum of 50 bacteriologically confirmed cases of pulmonary tuberculosis (Group I or Sm+), 50 serum from negative sputum smear tuberculosis (Group II or Sm-), 50 serum from extra-pulmonary tuberculosis (Group III or Ex-pul.) and 50 serum from cases of community acquired pneumonia (Group IV or CAP) collected from March 2011 to May 2012 were evaluated. The enzyme activity level was measured by Giusti method. The study was approved by the ethics committee of Arak University of Medical sciences. The data were analyzed using SPSS software. The data of quantitative variables are summarized as mean and range and Demographic data of subjects from different groups were compared by one-way analysis of variance (one way-ANOVA). Categorical variables were computed as frequency and percentage. The cutoff value of ADA was chosen according to a receiver operating characteristic (ROC) analysis. The positive predictive value, negative predictive value, sensitivity, specificity and likelihood ratio for positive and negative test were determined. The significance of association for categorical variables was estimated by Fisher's exact test. The differences in significance between continuous variables were compared by the Mann-Whitney u test and t-test. A two-tailed P-value of <0.05 was considered significant.

## Results

A total of 200 subjects were included in the study designed in four groups(Sm+,Sm-,Ex-pul. And CAP). 22(44%) males and 28(56%) females with a mean age of 52.96 ± 22.14 were diagnosed as group I, 29(58%) males and 21(42%) females with a mean age of 59.10 ± 18.29 were diagnosed as group II, 23(46%) males, 27(54%) females with a mean age of 56.14 ± 20.69 were diagnosed as group III and 26(52%) males, 24(48%) females with a mean age of 58.94 ± 17.58 were diagnosed as group IV. The mean value for serum ADA level that determined in Sm+, Sm&minus, Ex-pul. And CAP patients were 18.09 ± 6.64, 17± 5.10, 18.74± 3.45 and 15.55 ± 4.55 U/lit respectively.

In One-way Analysis of Variance (ANOVA) of mean differences of four groups the differences between group III (Ex-pul.) and group IV (CAP) was significant. (P-value =0.0115) (Table 1) The difference between the tuberculosis patient and non-tuberculosis patients with Mann-Whitney U test the differences between group I (Sm+) and group III with group IV (CAP) was significant. (P-value =0.0471 and p-value=0.0001) (Table 2) In our study the relationship between age, gender, new case or relapse, type of extra-pulmonary tuberculosis and degree of positivity of sputum smear with serum level of Ada did not exist (p-value>0.05).

**Table 1:** One-way Analysis of Variance (ANOVA) of four groups

Comparison	Mean differences	95%CI	P - value
Group I vs Group II	1.094	-1.539 - 3.727	P>0.05
Group I vs Group III	-0.6496	-3.283 - 1.984	P>0.05
Group I vs Group IV	2.534	-0.09961 - 5.167	P>0.05
Group II vs Group III	-1.744	-4.377 - 0.8896	P>0.05
Group II vs Group IV	1.440	-1.194 - 4.073	P>0.05
Group III vs Group IV	3.183	0.5500 - 5.816	0.0115

**Table 2:** Difference between the groups with Mann-Whitney test

	Sm+ vs CAP	Sm- vs CAP	Ex-pul. Vs CAP
Z	1.985	1.138	3.833
U	1538.0	1415.0	1806.0
P-value (two tailed)	0.0471	0.2553	0.0001

Considering the cutoff value of 24 U/l for ADA in group I defined the sensitivity, specificity, positive predictive value and negative predictive value 12%, 98%, 86%, and 56% respectively. group II defined the 6%, 98%, 75% and 51% and group III defined the 14%, 98%, 88% and 53% respectively. (Table 3)

**Table 3:** Diagnostic characteristics of serum ADA

	Sm+	Sm-	Ex-pul.
Sensitivity	12%	6%	14%
Specificity	98%	98%	98%
Positive PV	86%	75%	88%
Negative PV	53%	51%	53%
Positive LR	6	3	7
Negative LR	0.9	0.96	0.88
Accuracy	55%	52%	56%
Area under curve(ROC Analysis)	0.6263	0.6463	0.7354
Positive test Posterior probability	86%	86%	88%
Negative test Posterior probability	43%	47%	47%

## Discussion

In our study, no significant differences in age, sex, place of residence and compared the serum levels of ADA in four groups were not found significant differences. Several studies were carried out, have suggested the use of serum ADA levels for the diagnosis of pulmonary tuberculosis. Thora et al. [11] studied ADA levels of 100 newborn sera who were vaccinated with BCG showing a significant increase, indicating human cell-mediated immune response against mycobacterium antigens. Mishra et al. [12] evaluated serum ADA levels of 51 children with confirmed tuberculosis (pulmonary, peritoneal, meningeal, and bone), and 20 healthy controls showing significant increase in the first group with a p-value of <0.001. Collazos et al. [13] performed a prospective follow up study of 25 cases of pulmonary and pleural tuberculosis with a normal immune response for a period of 6 months after initiation of treatment. There was a significant decline in the serum ADA values during the first two months in the patients as a whole (P=0.04), followed by stabilization of the serum ADA activity. This decline was due to a marked decrease in the serum ADA activity in 13patients (52%) who had initial high levels of enzyme (p=0.03), whereas there were no changes in those patients with normal initial levels (p=0.27). Similar results were obtained by Ishii et al. [14] in Japan together with a direct association between serum ADA level and erythrocyte sedimentation rate. Joshaghani et al. [15] showed that the assessment of these enzymes in serum to some extend can be a useful method for differentiation of healthy subjects from respiratory disease, but these tests do not have enough sensitivity to assist in the diagnoses of tuberculosis patients from other respiratory diseases. Conde et al. [16] evaluated serum ADA in active pulmonary tuberculosis and other pulmonary infections and showed no significant difference between them. Their results were in disagreement with the report by Yasuhara et al. [17] in which serum ADA activity of children with active pulmonary tuberculosis was found to be significantly greater than those with bacterial or viral pneumonia. Serum activity of ADA has good specificity and positive predictive value. For several years, the ability to differentiate the diagnosis of PTB and non-PTB was an important issue for clinicians. Serum ADA activity is one such method, for which there have been varying results. Lamsal et al. [18] found a cut-off value of 25 U/L, a test sensitivity of 72.41%, and a specificity of 81.53%. Kuyucu et al. [19] reported a serum ADA level of greater/equal to 53.76 U/L, a sensitivity of 100%, and a specificity of 90.7%, while indicating a positive predictive value of 58.8%, and a negative predictive value of 100% in children with TB. Bhargave et al. [20] and Al-Shammary et al. [21] reported the cut-off value of serum ADA levels in tuberculosis patients as 78.12 IU/L and 32.8 U/L, respectively

## Conclusion

In our study, the optimum cut-off point of ADA for distinguishing tuberculosis and non-tuberculosis subjects was found to be >24 U/L using the ROC curve and has low sensitivity, high specificity in tuberculosis patients that indicated this test do not have enough sensitivity to assist in the diagnoses of tuberculosis patients from other respiratory diseases and not evaluated perform well enough to replace sputum smear microscopy. Thus this test have little role in the diagnosis of pulmonary tuberculosis. Although determination of ADA is not costly or time consuming and is relatively easy to do but ADA estimation should not be done routinely. We concluded that serum ADA activity is not a useful test to differentiate tuberculosis from other respiratory diseases, And Can only be an auxiliary test particularly if the diagnosis of tuberculosis is in doubt.
